# Adeno-associated virus-based caveolin-1 delivery via different routes for the prevention of cholesterol gallstone formation

**DOI:** 10.1186/s12944-022-01718-7

**Published:** 2022-10-27

**Authors:** Sha Li, Hongtan Chen, Xin Jiang, Fengling Hu, Yiqiao Li, Guoqiang Xu

**Affiliations:** 1 Department of Gastroenterology, the First Affiliated Hospital, Zhejiang University School of Medicine, 310006 Hangzhou, Zhejiang China; 2 Urology & Nephrology Center, Department of Nephrology, Zhejiang Provincial People’s Hospital and Hangzhou Medical College Affiliated People’s Hospital, 158 Shangtang Road, 310014 Hangzhou, Zhejiang China

**Keywords:** Cholesterol gallstone disease, caveolin-1, Adenosine monophosphate-activated protein kinase, mucin-1, mucin-5ac

## Abstract

**Background:**

Hepatic caveolin-1 (CAV1) is reduced in cholesterol gallstone disease (CGD). Mice with CAV1 deficiency were prone to develop CGD. However, it remains unknown whether restored hepatic CAV1 expression prevents the development of CGD.

**Methods:**

C57BL/6 mice were injected with adeno-associated virus 2/8 (AAV2/8) vectors carrying the CAV1 gene (^AAV2/8^CAV1) via intravenous (i.v.) or intraperitoneal (i.p.) route and then subjected to a lithogenic diet (LD) for 8 weeks. Uninjected mice were used as controls. The functional consequences of rescuing CAV1 expression by either i.v. or i.p. ^AAV2/8^CAV1 treatment for CGD prevention and its subsequent molecular mechanisms were examined.

**Results:**

CAV1 expression was reduced in the liver and gallbladder of LD-fed CGD mice. We discovered that ^AAV2/8^CAV1 i.p. delivery results in higher transduction efficiency in the gallbladder than tail vein administration. Although either i.v. or i.p. injection of ^AAV2/8^CAV1 improved liver lipid metabolic abnormalities in CGD mice but did not affect LD feeding-induced bile cholesterol supersaturation. In comparison with i.v. administration route, i.p. administration of ^AAV2/8^CAV1 obviously increased CAV1 protein levels in the gallbladder of LD-fed mice, and i.p. delivery of ^AAV2/8^CAV1 partially improved gallbladder cholecystokinin receptor (CCKAR) responsiveness and impeded bile cholesterol nucleation via the activation of adenosine monophosphate-activated protein kinase (AMPK) signaling, which induced a reduction in gallbladder mucin-1 (MUC1) and MUC5ac expression and gallbladder cholesterol accumulation.

**Conclusion:**

CGD prevention by i.p. ^AAV2/8^CAV1 injection in LD-fed mice was associated with the improvement of gallbladder stasis, which again supported the notion that supersaturated bile is required but not sufficient for the formation of cholesterol gallstones. Additionally, AAV treatment via the local i.p. injection offers particular advantages over the systemic i.v. route for much more effective gallbladder gene delivery, which will be an excellent tool for conducting preclinical functional studies on the maintenance of normal gallbladder function to prevent CGD.

**Supplementary Information:**

The online version contains supplementary material available at 10.1186/s12944-022-01718-7.

## Introduction

Caveolae, which play an important role in endocytosis and signal transduction, are presented on the plasma membrane as a small, flask-shaped pit [1]. Caveolin-1 (CAV1) is an essential component of caveolae, which is also involved in vesicular trafficking, lipid and cholesterol metabolism, and signaling cascades [1]. CAV1 can traffic between the cytoplasm and membrane, contributing to the maintenance of intracellular cholesterol homeostasis and the transportation of extracellular cholesterol [2]. Intracellular cholesterol also affects caveolae density, CAV1 expression, and redistribution [2]. Despite the fact that CAV1 is found in both the basolateral and apical plasma membranes of murine liver cells, caveolae form only in the basolateral plasma membranes[3]. CAV1 can also be detected in endosomes, lipid droplets, mitochondria, endoplasmic reticulum, and other intracellular compartments, suggesting that CAV1 may act in a caveolae-independent manner in the liver [3]. CAV1 is thought to play a key role in cholesterol gallstone disease (CGD), nonalcoholic fatty liver disease (NAFLD), and other diseases associated with high cholesterol levels [2–6].

CGD is common. Although the exact molecular mechanism remains unclear, excessive accumulation of biliary cholesterol and abnormal cholesterol nucleation time (NT) are considered relevant factors for CGD development [7]. In the murine CGD model, we observed an increase in the secretion of biliary cholesterol, a decrease in the synthesis of bile acids, and an increase in the bile cholesterol saturation index (CSI)[6]. Using CAV1 knockout mice can significantly increase the incidence of CGD after 4 weeks of a lithogenetic diet [6]. However, it is still not clear whether CAV1 overexpression slows the formation of CGD.

Adeno-associated virus (AAV) is a member of the Parvoviridae family, emerging as a small, membraneless virus with icosahedral structures [8]. These AAV constructs range in diameter from 20 to 26 nm and contain linear single-stranded DNA genomes ranging from 4.7 to 6 kb. AAV is widely acknowledged as a safe and effective form of gene delivery due to its low immunogenicity, high infection capacity, and long-term steady expression of genes. The systemic intravenous (i.v.) or local intraperitoneal (i.p.) injection of AAV-mediated gene delivery is a widely used approach for overexpression of a specific gene in the liver of mice [9]. However, it has been demonstrated before that i.p. injection of AAV transduces pancreatic cells more efficiently than i.v. injection [10, 11]. In addition, is there a difference in gallbladder-directed AAV transduction efficiency between i.v. and i.p. routes? This question remains to be determined.

Through this study, we aimed to elucidate the effective method of gallbladder CAV1 overexpression via AVV gene delivery by comparing two different delivery routes (i.v. vs. i.p.). The effects of CAV1 on the prevention of CGD should be investigated.

## Materials and methods

### Animals, diet and drug treatment

All animal experiments were conducted following the Chinese Ministry of Health national guidelines for the housing and care of laboratory animals and were approved by the Animal Committee of Zhejiang University (No. 2020 − 1288). Each mouse experiment was performed in triplicate. Three-month-old male C57BL/6 mice (total number of mice = 84) were purchased from the Model Animal Research Center of Nanjing University (Nanjing, China). The numbers of mice/group are given in the figure legends. All mice were housed in a pathogen-free animal facility under controlled conditions of humidity (55 ± 5%), lighting (12-hour light/dark cycle), and temperature (23 °C) and were given diet and water *ad libitum*.

The murine CAV1 cDNA sequences were obtained from the PubMed database and were synthesized by Shanghai GeneChem Co., Ltd. (Shanghai, China), which were then cloned and packaged as AAV2 inverted terminal repeat DNA combined with the AAV8 capsid (AAV2/8) virus. To address the hypothesis that CAV1 gene overexpression by using AAV2/8 gene delivery (^AAV2/8^CAV1) can prevent CGD, mice received an i.v. or i.p. injection of ^AAV2/8^CAV1 at a dose of 1 × 10^11^ viral genomes (vg)/animal and were then fed a lithogenic diet (LD, TD.90,221, containing 15.8% fat, 1.25% cholesterol, and 0.5% sodium cholate; Harlan Teklad Custom Research, Livermore, CA, USA) or a control chow diet (TD.2918; Harlan Teklad Custom Research, Livermore, CA, USA) for 8 weeks. Mice were then euthanized for collection of ileum, liver, and gallbladder tissues. The samples were frozen in liquid nitrogen and stored at − 80 °C until analysis. For drug treatment, the adenosine monophosphate-activated protein kinase (AMPK) inhibitor compound c (866405-64-3, 10 mg/kg, i.p. injection once a day; Sigma, Ronkonkoma, NY, USA) was given at the beginning of the LD, and the drug treatments continued until the completion of the experiments.

### Examination of AAV2/8CAV1 transduction efficiency

Frozen Sect. (5 μm thick) of the gallbladder, ileum, and liver from mice injected with or without ^AAV2/8^CAV1 (8 weeks postinjection) were used for immunofluorescence staining. Serial sections were incubated with a mixture of primary antibodies against CAV1 (rabbit monoclonal, E249, 1:500; Abcam, UK) and β-actin (mouse monoclonal 8226, 1:1000; Abcam, UK). After three washes, the mixture of secondary antibodies (cy3-conjugated goat anti-mouse IgG 2,338,714 and fluorescein isothiocyanate–conjugated goat anti–rabbit IgG 2,337,972; Jackson ImmunoResearch Laboratories) was overlaid. Cell nuclei were counterstained with 2-(4-amidinophenyl)-6-indolecarbamidine (DAPI; 300 nM, D9542; Sigma, Ronkonkoma, NY, USA).

Fluorescent images of cryosections were recorded using an Olympus DP70 digital camera coupled to an Olympus IX71 inverted microscope (Tokyo, Japan). Fluorescence expression efficiency was measured using ImageJ software. The fluorescence intensities of CAV1 expression areas were normalized to the fluorescence intensities of β-actin-stained areas. The double-stained area of CAV1/β-actin was counted in five different arbitrary areas from three independent experiments. Total intensities of 100 cells from multiple areas were accumulated and considered as the value of CAV1 and β-actin expression in gallbladder, ileum, and liver from one mouse.

To investigate the efficiency of ^AAV2/8^CAV1 transduction, compared with the i.v. and i.p. routes, total DNA was extracted from the gallbladder, ileum and liver tissues of mice with a DNAeasy Blood and Tissue kit (Qiagen 69504; Valencia, CA, USA), and vector genome copy number was determined with a 7900 HT real-time PCR system (Applied Biosystems; Foster City, CA, USA). TaqMan assays for viral vector genome copy number were developed using primers and probes for the AAV2/8 vector bearing a small chicken β-actin promoter region. The forward primer was 5’-TCTGCTTCACTCTCCCCATCTC-3’. The reverse primer was 5’-CCATCGCTGCACAAA- ATAATTAAA-3’. The fluorescent probe was 6-carboxyfluorescein-CCCCCTCCCCACCCCC-AATT. The AAV2/8 vector genome copy number is normalized as the viral genome copy number per µg of total genomic DNA.

### LD consumption

After being fed the LD for 8 weeks, the mice were singly housed. Food pellets were weighed (grams) per day in the morning over a 3-day LD consumption period, and the amount of food left in the cages was subtracted from the initially recorded amount.

### Measurement of fecal cholesterol excretion

After being fed the LD for 8 weeks, the mice were singly housed. After a 3-day fecal collection, the mice were weighed, and the feces were dried in a 70 °C vacuum oven, weighed, and crushed into a fine powder. A measured mass (50 mg) of feces was placed into a glass tube containing 103 µg of 5α-cholestane as an internal standard. The feces were saponified, and the neutral lipids were extracted with hexane. Mass analysis of the extracted neutral sterols was conducted by the DIAN Diagnostics Laboratory (Hangzhou, China) using gas‒liquid chromatography. Fecal cholesterol mass represents the sum of cholesterol and its derivatives (coprostanol and coprostanone) in each sample. Fecal cholesterol excretion was expressed as µmol/day/100 g body weight.

### Biochemical analysis of liver and gallbladder tissues and gallbladder bile

After being fed LD for 8 weeks, the hepatic tissues (150 mg) of mice were homogenized, and the triglyceride, cholesterol, and free fatty acid (including palmitic, stearic, oleic, linoleic and arachidonic acid proportions) levels were examined by the DIAN Diagnostics Laboratory (Hangzhou, China).

The phospholipid, cholesterol and triglyceride levels in the gallbladder bile of 8-week LD-fed mice were measured by the DIAN Diagnostics Laboratory (Hangzhou, China). Total bile salts were quantified with the 3α-hydroxysteroid dehydrogenase enzymatic method. The cholesterol saturation index (CSI) was calculated using Carey’s critical table.

### Microscopic examination of cholesterol crystals

After 8 weeks of LD, mice were fasted overnight and euthanized, and then the intact gallbladders were harvested. Gallbladder bile was spread on glass slides and examined with a Leica DM5000 polarized microscope for the detection of cholesterol crystals.

### Examination of gallbladder contraction

After being fed the LD for 8 weeks, the contractility of the gallbladder of mice was measured using a MyoMED myograph system (MED Associates, USA). Chow-fed mice were used as a control. After anesthetized euthanasia, the gallbladders were removed, and the gallbladder strips (measuring from 3 to 10 mm) were cut through the whole wall. The silk threads were attached to each end of the strips. Each strip was mounted in a tissue bath (15-mL volume) containing aerated (5% CO2/95% O2) physiological saline solution (containing 119 mM NaCl, 4.7 mM KCl, 24 mM NaHCO3, 1.2 mM KH2PO4, 2.5 mM CaCl2, 1.2 mM MgSO4, and 11 mM glucose; pH 7.4) at 37 °C. These gallbladder strips were placed under an initial resting tension equivalent to a 5 mN load and allowed to equilibrate for 60 min, with solution changes every 15 min. Agonist responses were obtained by applying the neurotransmitter cholecystokinin (AS20741, 10 nM, AnaSpec, Fremont, USA) or the muscarinic agonist methacholine (carbachol 51-83-2, 0.1 ~ 30 µM, applied cumulatively; Sigma, Ronkonkoma, NY, USA) directly into the tissue bath. Responses were normalized to the wet weight of the tissue.

### Quantitative real-time polymerase chain reaction (qRT‒PCR) assays

qRT‒PCR was performed using total RNA extracted from ileum, liver, and gallbladder tissues of chow-fed and LD-fed mice (after an 8-week feeding period). All primer sequences used are listed in Supplementary Table 1. Expression data were normalized to the expression of 18 S RNA.

### Western blotting

The liver and gallbladder tissues collected from 8-week LD-fed mice were lysed with NE-PER™ nuclear and cytoplasmic extraction reagents (78,833, Thermo Fisher, Waltham, MA, USA) and complete protease inhibitor (11,697,498,001, Roche, Basel, Switzerland) on ice. For immunoblotting, the lysate proteins were resolved by SDS‒PAGE and blotted onto nitrocellulose membranes to test the binding of the antibodies (the antibodies used are listed in Supplementary Table 2). Original western blotting data are shown in Supplementary Figs. 1–3.

### Transfection of HepG2 cells and luciferase reporter assay

On Day 0, human liver HepG2 cells (HB-8065; ATCC, Manassas, VA, USA) were plated at a density of 5 × 10^4^ cells per well in 24-well plates in Eagle’s Minimum Essential Medium (ThermoFisher, Waltham, MA, USA) supplemented with 10% fetal bovine serum (FBS; ThermoFisher, Waltham, MA, USA) and incubated at 37 °C in a 5% CO_2_ incubator. On Day 1, the cells were washed with 0.5 mL phosphate-buffered saline (PBS), and 0.5 mL fresh Eagle’s minimum essential medium was added to each well before transfection. A 201 bp DNA fragment from intron 2 of the human ATP binding cassette subfamily G member 5 (ABCG5) gene (Homo sapiens Chr2, NC_000002.12: 43,836,293 ~ 43,836,093) [12], a 210 bp DNA fragment from intron 3 of the human ATP binding cassette subfamily G member 8 (ABCG8) gene (Homo sapiens Chr2, NC_000002.12: 43,848,694 ~ 43,848,903) [12], a 380 bp DNA fragment from the intragenic region of the human ABCG5/ABCG8 gene (Homo sapiens Chr2, NC_000002.12: 443,839,056 ~ 43,838,677, in either the ABCG5 or ABCG8 orientation) [12] and a 420 bp DNA fragment from the fragment of the human sterol-regulatory-element-binding protein-1c (SREBP1c) promoter (Homo sapiens Chr17, NC_000017.11: 17,824,067 ~ 17,823,648) [13] were synthesized by Generay Biotech (Generay Biotech, Shanghai, China) and inserted into a pGL3 firefly luciferase reporter vector (Promega, Madison, WI, USA). The pGL3 plasmid containing a 380 bp DNA fragment from the intragenic region of the human ABCG5/ABCG8 gene (in either the ABCG5 or ABCG8 orientation) served as a template to generate a GATA-mutated binding site (AGGCCG) construct, in which putative GATA binding sites were mutated (Homo sapiens Chr2, NC_000017.11: 43,838,839 ~ 43,838,834; AGATAA) [12]. Mutations were created by site-directed mutagenesis using the GeneArt™ Site-Directed Mutagenesis System (ThermoFisher A13282, Waltham, MA, USA). The mutation was sequence verified. Then, HepG2 cells were cotransfected with these above plasmids (0.25 µg) and a control plasmid pCMV-β-Gal vector (10 ng; Promega, Madison, WI, USA) containing the β-galactosidase reporter gene by using Lipofectamine 2000 (ThermoFisher, Waltham, MA, USA) transfection reagent. Each transfection was performed in triplicate wells. Six hours after transfection, the cells were washed with 0.5 mL PBS, switched with Eagle’s minimum essential medium supplemented with 10% FBS and 1 µM T0901317 (LXR agonist, Sigma, Ronkonkoma, NY, USA) plus 0.5 mM metformin (AMPK agonist, Sigma, Ronkonkoma, NY, USA), and incubated for 16 h at 37 °C and 5% CO_2_. On Day 2, the cells were washed with 0.5 mL PBS, and firefly luciferase activities were measured using the Luciferase Assay System (Promega, Madison, WI, USA) and TD-20/20 Luminometer (Turner Designs, San Jose, CA, USA). β-galactosidae activity was measured by the Galacto-Light β-Galactosidase Reporter Gene Assay System (ThermoFisher, Waltham, MA, USA). Firefly luciferase activities in the transfected lysates were normalized to β-galactosidase activity in the same tube.

### Statistical analyses

Data are expressed as the means ± standard deviations (SD). Statistical analysis was performed using Prism (GraphPad Software, San Diego, CA, USA). Data normality was determined by using the Shapiro‒Wilkes test. The differences in the incidence of CGD in mice were determined by Fisher’s exact test. The statistical analysis between two independent groups with normal distribution was determined by using Student’s t test, while the Mann‒Whitney test was used to compare nonnormal data. For more than two normally distributed groups, statistical comparisons were made by one-way analysis of variance (ANOVA, equal variance between groups) or Welch’s ANOVA (unequal variance between groups) with pairwise comparisons using Benjamini‒Hochberg corrections. Kruskal‒Wallis nonparametric ANOVA with Benjamini‒Hochberg correction was used to compare more than two samples with nonnormal distributions. *P* < 0.05 was considered significant.

## Result

### Intraperitoneal delivery leads to efficient gallbladder CAV1 gene transfer in mice.

**Fig. 1:**
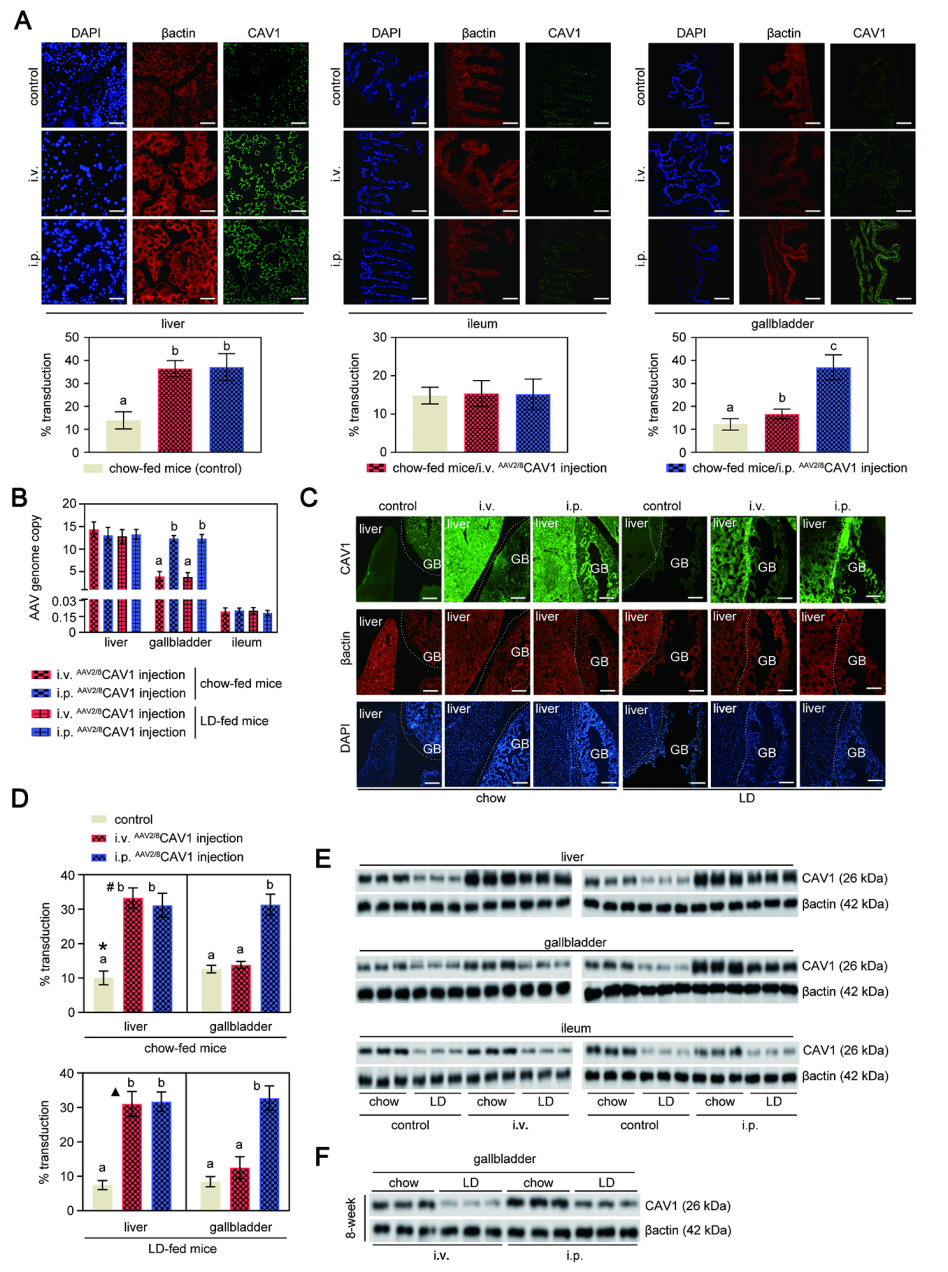
**The i.p. injection improves the delivery of** ^**AAV2/8**^ **CAV1 to the mouse gallbladder compared with i.v. injection** Mice were injected (either via the i.v. or i.p. route) with or without ^AAV2/8^CAV1 at 1 × 10^11^ vg/animal and then assigned to chow or LD (8-week) The letters on each bar are provided for statistical purposes, and different letters indicate significance (*P* < 0.05). If there are no significant differences between two bars, they have the same letter. The individual *P* values are described in Supplementary Table 3. A. Serial sections of frozen (5 μm) liver, gallbladder, and ileum tissues collected from mice were stained for β-actin (red) to quantify cell-specific CAV1 (green) transduction percentages. Representative images are shown. Blue: DAPI staining for nuclei. Scale bar: 20 μm. Quantification of liver, gallbladder, and ileum transduction was determined by the percentage of CAV1 staining-positive area. The fluorescence intensities of CAV1 expression areas were normalized to the fluorescence intensities of the β-actin-stained area. The results in the lower panel are shown as the mean ± SD (three sections from each animal were analyzed). n = 13 for each group, mice with either i.v. or i.p. administration of ^AAV2/8^CAV1; n = 5 for control (uninjected) mice. B. qRT‒PCR analysis of AAV genomes in liver, gallbladder, and ileum collected from chow-fed (n = 9 each group, mice with either i.v. or i.p. administration of ^AAV2/8^CAV1) or LD-fed (n = 13 for each group, mice with either i.v. or i.p. administration of ^AAV2/8^CAV1) mice. C. Cryosections (5 μm) of liver and gallbladder tissues collected from mice were stained for β-actin (red) to quantify cell-specific CAV1 (green) transduction percentages. Representative images are shown. Blue: DAPI staining for nuclei. Scale bar: 100 μm. GB, gallbladder. D. Quantification of liver and gallbladder transduction was determined by the percentage of CAV1 staining-positive area. The fluorescence intensities of CAV1 expression areas were normalized to the fluorescence intensities of β-actin-stained areas under the same fluorescence microscopic field. The results in the right panel are shown as the mean ± SD (three sections from each animal were analyzed). n = 13 for each group, mice with either i.v. or i.p. administration of ^AAV2/8^CAV1; n = 5 for control (uninjected) mice. *, *P* = 0.047 for the fluorescence intensities of CAV1 expression areas in liver versus GB in chow-fed control mice; #, *P* = 5.7e-14 for the fluorescence intensities of CAV1 expression areas in liver versus GB in chow-fed i.v. ^AAV2/8^CAV1-injected mice; ▲, *P* = 5.95e-13 for the fluorescence intensities of CAV1 expression areas in liver versus GB in LD-fed i.v. ^AAV2/8^CAV1-injected mice. E. Western blotting analysis of CAV1 protein expression in the liver, gallbladder, and ileum of chow-fed or LD-fed mice. β-actin was used as a loading control. The data are representative of three samples for each protein. i.v., ^AAV2/8^CAV1 injection (1 × 10^11^ vg/mice) via the i.v. route; i.p., ^AAV2/8^CAV1 injection (1 × 10^11^ vg/mice) via the i.p. route. F. Western blotting analysis for the comparison of CAV1 protein expression in the gallbladder of chow-fed or LD-fed mice between i.v. (1 × 10^11^ vg/mouse) and i.p. (1 × 10^11^ vg/mouse) routes of administration of ^AAV2/8^CAV1. β-actin was used as a loading control. The data are representative of three samples for each protein.

Since CAV1 is widely distributed in gallbladder, intestine, and liver tissues (https://www.proteinatlas.org/ENSG00000105974-CAV1/tissue), we evaluated the effect of different routes of delivery and AAV-mediated CAV1 gene transduction in those three tissues. We compared i.p. and i.v. ^AAV2/8^CAV1 delivery in adult mice. Briefly, 1 × 10^11^ vg per mouse of ^AAV2/8^CAV1 was administered to 8-week-old mice, either by i.v. or i.p. injection. At 8 weeks postinjection, the liver, gallbladder, and ileum tissues were collected to analyze CAV1 expression. No significant differences were observed in CAV1 expression or the AAV vector genome copy number in the livers of mice between i.v. and i.p. injection **(**Fig. 1 A-D**)**. We found that i.p. ^AAV2/8^CAV1 delivery increased transduction efficiency in the gallbladder compared with i.v. injection **(**Fig. 1 A-D**)**. In addition, we confirmed that ^AAV2/8^CAV1 did not transduce in the ileum (Fig. 1 A **and B**) at a high frequency (< 3% CAV1 gene transduction efficiency), which is consistent with prior findings [14]. Western blotting further verified these results **(**Fig. 1E **and F).**

### ***Intraperitoneal administration of***^***AAV2/8***^***CAV1 prevents CGD, regardless of changes in biliary CSI.***

**Fig. 2:**
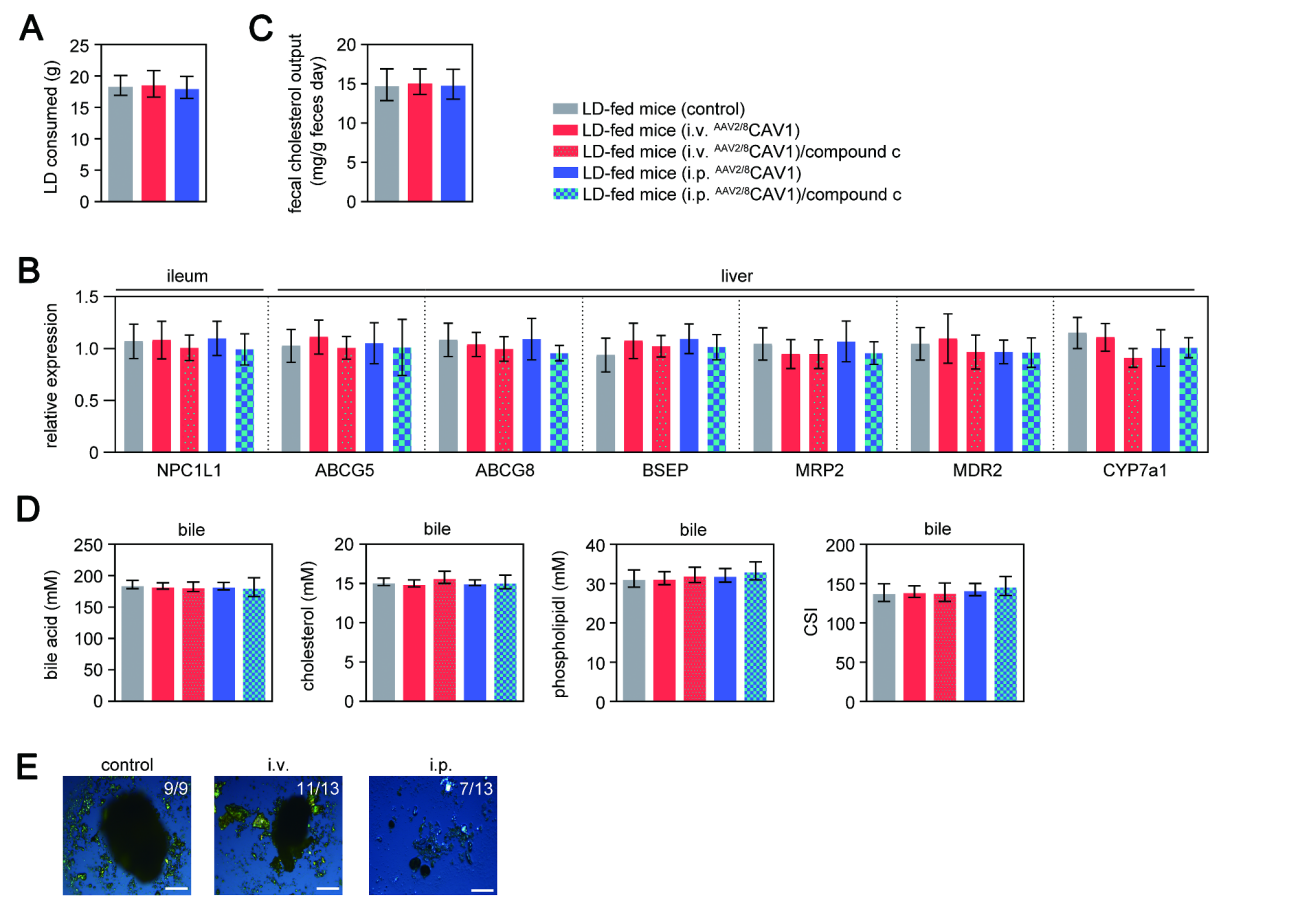
^**AAV2/8**^ **CAV1 treatment via the i.p. route can partially prevent CGD in LD-fed mice through the biliary CSI-independent pathway** Mice were injected (either via the i.v. or i.p. route) with or without ^AAV2/8^CAV1 at 1 × 10^11^ vg/animal and then fed LD following PBS or compound c treatment (8 weeks). n = 13 for each group, LD-fed mice with either i.v. or i.p. administration of ^AAV2/8^CAV1 with or without compound c treatment (10 mg/kg, i.p. injection once a day); n = 9 for LD-fed mice (control) with neither ^AAV2/8^CAV1 administration nor compound c treatment The letters on each bar are provided for statistical purposes, and different letters indicate significance (*P* < 0.05). If there are no significant differences between two bars, they have the same letter. The individual *P* values are described in Supplementary Table 3. A. Lithogenic diet mass consumed by each group of mice. B. qRT‒PCR analysis of the liver, gallbladder, and ileum messenger RNA expression of cholesterol, phospholipid transporters, and bile acid transporters in each group of mice. 18 S rRNA was used as an internal control. Data represent the mean ± SD. C. Twenty-four-hour cumulative fecal samples collected from each group of mice were pooled and measured for total cholesterol contents. D. Biliary concentrations of cholesterol, phospholipids, bile acid, and CSI in each group of mice. E. Polarizing light microscopy examination of cholesterol crystals in the gallbladder of each group of mice. i.v., ^AAV2/8^CAV1 injection (1 × 10^11^ vg/mice) via the i.v. route; i.p., ^AAV2/8^CAV1 injection (1 × 10^11^ vg/mice) via the i.p. route.

CAV1 is considered to participate in the regulation of hepatic lipid accumulation and cholesterol metabolism, thus playing an important role in the pathogenesis of diseases related to aberrant triglyceride or cholesterol metabolism[3]. Therefore, we examined the concentrations of bile acid, lecithin, and cholesterol in gallbladder bile of chow-fed or LD-fed mice after injection of ^AAV2/8^CAV1. The levels of CAV1 protein in the liver and gallbladder of control (noninjected) mice decreased after 8 weeks of LD feeding (Fig. 1E **and F**). Either i.v. or i.p. administration of ^AAV2/8^CAV1 elevated the hepatic CAV1 expression collected from 8-week LD-fed mice (Fig. 1E). In addition, the CAV1 concentration in the gallbladder was higher after i.p. injected mice than in i.v. injected mice and control mice (Fig. 1E **and F**). CAV1 overexpression did not alter the feeding behavior of the mice (Fig. 2 A). After LD feeding, the intestinal expression of niemann-pick disease, type C1-like intracellular cholesterol transporter 1 (NPC1L1) and the fecal contents of cholesterol were also similar between control mice and ^AAV2/8^CAV1-treated mice (Fig. 2B **and C**), which implied that CAV1 overexpression did not change oral cholesterol uptake.

CGD results from the imbalance between bile acids, cholesterol, and phospholipids in gallbladder bile[4, 7]. The canalicular efflux of cholesterol, bile acids, and phospholipids mediated by ABCG5/G8, bile salt export pump (BSEP), multidrug resistance protein 2 (MRP2) and multidrug resistance protein 2 (MDR2), respectively, which directly regulate bile cholesterol saturation, was similar between control mice and ^AAV2/8^CAV1-treated mice after LD feeding (Fig. 2B). There were no significant differences in the contents of biliary bile acids, cholesterol, phospholipids or biliary CSI between ^AAV2/8^CAV1-treated or control mice **(**Fig. 2D**)**. However, the CGD prevalence **(**Fig. 2E**)** was similar between the control and i.v. ^AAV2/8^CAV1-treated mice after 8 weeks of LD feeding (9/9 control mice versus 11/13 i.v. ^AAV2/8^CAV1 treated mice, *P* = 0.49, by Fisher’s exact test), while which was detected as a lower incidence in LD-fed mice with i.p. ^AAV2/8^CAV1 administration (9/9 control mice versus 7/13 i.p. ^AAV2/8^CAV1-treated mice, *P* = 0.04, by Fisher’s exact test).

### ***AMPK transactivates ABCG5/G8 gene expression to increase biliary cholesterol output.***

**Fig. 3:**
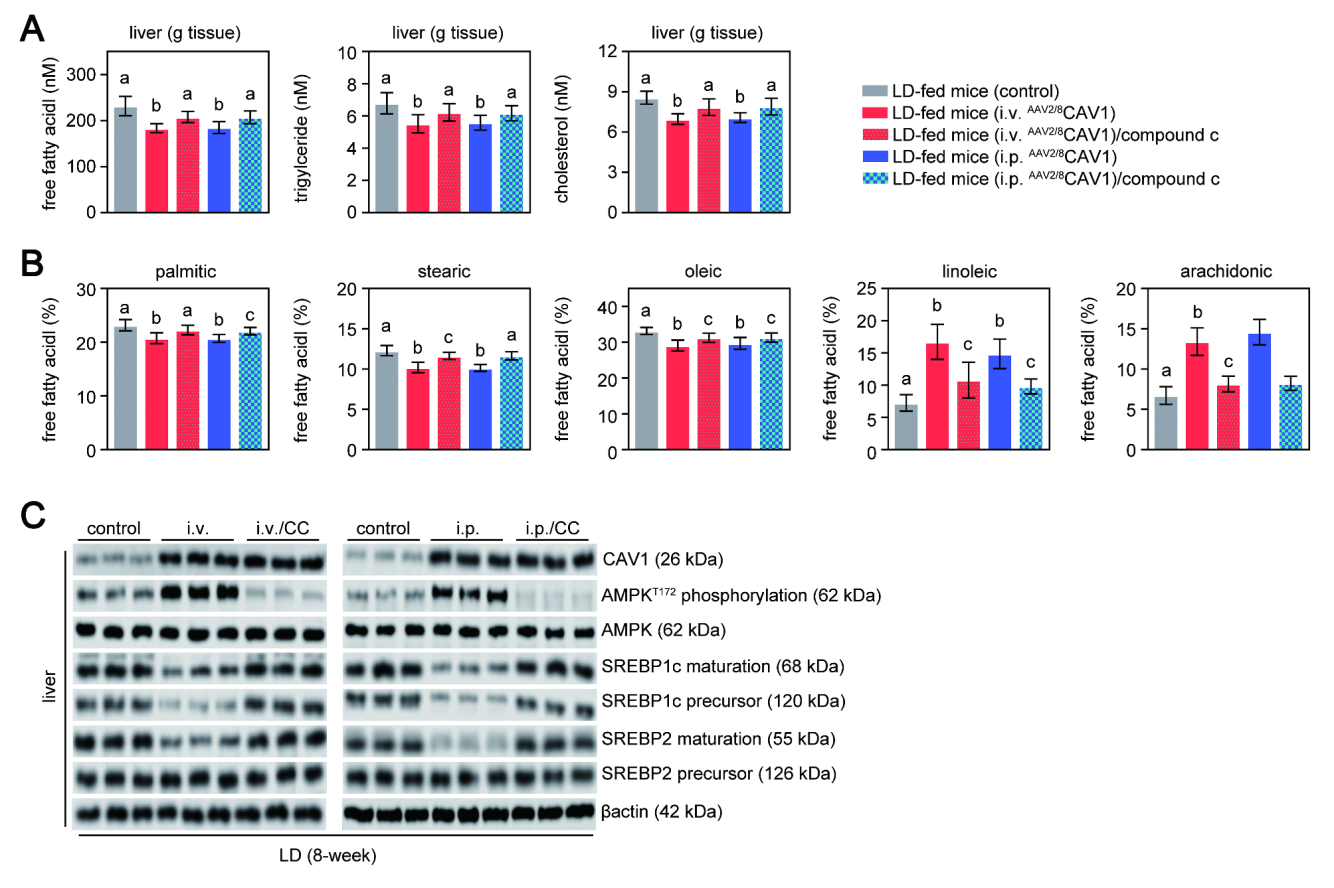
**AMPK inhibition hampered the protective effect of** ^**AAV2/8**^ **CAV1 treatment on LD-induced hepatic lipid accumulation** Mice were injected (either via the i.v. or i.p. route) with or without ^AAV2/8^CAV1 at 1 × 10^11^ vg/animal and then fed LD. n = 13 for each group, LD-fed mice with either i.v. or i.p. administration of ^AAV2/8^CAV1 with or without compound c treatment (10 mg/kg, i.p. injection once a day); n = 9 for LD-fed mice (control) with neither ^AAV2/8^CAV1 administration nor compound c treatment The letters on each bar are provided for statistical purposes, and different letters indicate significance (*P* < 0.05). If there are no significant differences between two bars, they have the same letter. The individual *P* values are described in Supplementary Table 3. A. Quantitation of free fatty acids, triacylglycerol, and cholesterol in the liver of each group of mice. B. Proportion of each liver free fatty acid profile (palmitic acid, stearic acid, oleic acid, linoleic acid, and arachidonic acid) in each group of mice. C. Western blotting analysis of AMPK (and its phosphorylation), CAV1, and SREBP1c protein expression in the liver and gallbladder of each group of mice. β-actin was used as a loading control. Data are representative of three samples for each protein. T172, the activation marker of AMPK phosphorylation at threonine 172; i.v., ^AAV2/8^CAV1 injection (1 × 10^11^ vg/mice) via the i.v. route; i.p., ^AAV2/8^CAV1 injection (1 × 10^11^ vg/mice) via the i.p. route; CC, compound C injection (10 mg/kg/day) via the i.p. route.

Our previous work showed that global CAV1 deficiency promotes CGD via the progression of hepatic lipid metabolism dysfunction and the upregulation of liver X receptor (LXR)-ABCG5/G8 signaling [6]. Here, we found that the levels of hepatic triglycerides and cholesterol were much lower in LD-fed mice given ^AAV2/8^CAV1 via the i.v. or i.p. than in control mice **(**Fig. 3 A**)**.

This has been borne out in several studies [15–19], and we have proven that CAV1 overexpression by ^AAV2/8^CAV1 treatment prevented LD feeding-induced hepatic steatosis and abnormal lipid metabolism by reducing SREBP1c expression via AMPK pathway activation[20] **(**Fig. 3B**)**. AMPK also suppressed hepatic de novo cholesterol synthesis by inhibiting hepatic sterol-regulatory-element-binding protein-2 (SREBP2) maturation [20] **(**Fig. 3B**)**, which may explain why hepatic cholesterol levels decreased but biliary cholesterol levels remained unchanged **(**Figs. 2D and 3 A**)**. Treatment with compound c (AMPK inhibitor) removed the protective effects of ^AAV2/8^CAV1 treatment on aberrant hepatic lipid metabolism **(**Fig. 3 A **and B)**. Additionally, hepatic principal bile acid synthesis enzyme cytochrome P450 7A1 (CYP7a1) expression was comparable between control mice and ^AAV2/8^CAV1-treated mice after LD feeding (Fig. 2B), although a previous study has shown that AMPK limits the conversion of cholesterol to bile acids by suppressing the hepatic expression of CYP7a1 in human HepG2 cells [21].

**Fig. 4:**
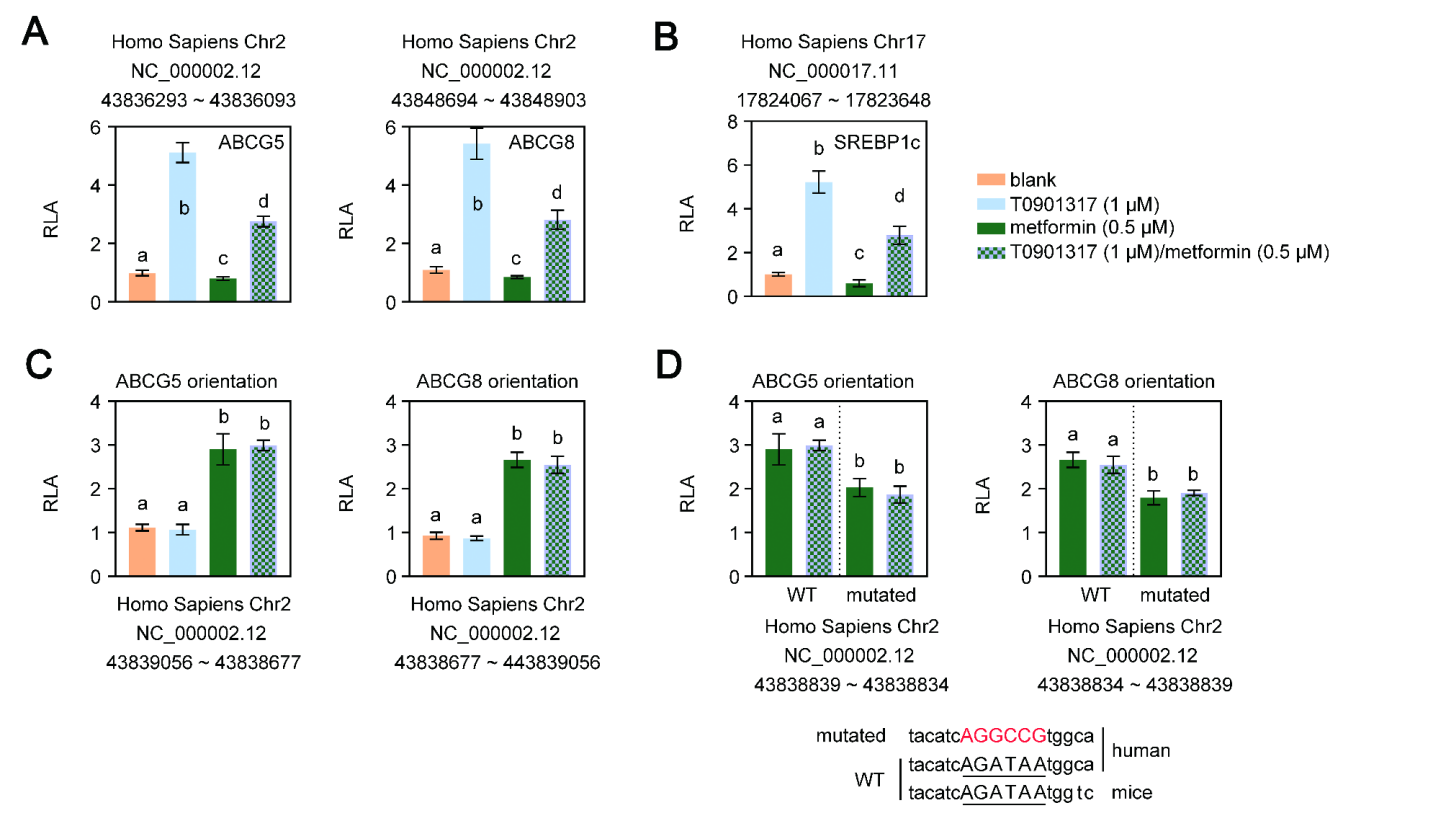
**AMPK and LXR transactivate the human ABCG5/G8 gene at different sites** Data represent the mean ± SD of fold-independent experiments, expressed as fold-change vs. untreated cells The letters on each bar are provided for statistical purposes, and different letters indicate significance (*P* < 0.05). If there are no significant differences between two bars, they have the same letter. The individual *P* values are described in Supplementary Table 3. RLA, relative luciferase activity. A and B. HepG2 cells were cotransfected with the pGL3 reporter containing a DNA fragment from intron 2 of the human ABCG5 gene (A, left panel), a DNA fragment from intron 3 of the human ABCG8 gene (A, right panel), an intragenic region (in either the ABCG5 or ABCG8 orientation) of the human ABCG5/ABCG8 gene (B), and a control plasmid (the plasmid containing the β-galactosidase reporter gene). Six hours after transfection, cells were treated with 1 µM T0901317 (LXR agonist) plus 0.5 mM metformin (AMPK agonist). Untreated cells were used as a control. Twenty-four hours later, the cells were washed with 0.5 mL PBS, and luciferase and β-galactosidase activities were quantified using a luciferase assay kit. Normalized firefly luciferase activity by β-galactosidase activity without treatment was set as 1. C. HepG2 cells were cotransfected with the pGL3 reporter containing a 420 bp DNA fragment from the fragment of the human SREBP1c promoter and a control plasmid (the plasmid containing the β-galactosidase reporter gene). Six hours after transfection, cells were treated with 1 µM T0901317 (LXR agonist) plus 0.5 mM metformin (AMPK agonist). Untreated cells were used as a control. Twenty-four hours later, the cells were washed with 0.5 mL PBS, and luciferase and β-galactosidase activities were quantified using a luciferase assay kit. Normalized firefly luciferase activity by β-galactosidase activity without treatment was set as 1. D. HepG2 cells were cotransfected with the pGL3 reporter containing an intragenic region of the human ABCG5/ABCG8 gene (in either the ABCG5 or ABCG8 orientation) with a GATA-mutated binding site, or a wild-type intragenic region of the human ABCG5/ABCG8 gene (in either the ABCG5 or ABCG8 orientation), and a control plasmid (the plasmid containing the β-galactosidase reporter gene). Six hours after transfection, cells were treated with 0.5 mM metformin (AMPK agonist) or plus 1 µM T0901317 (LXR agonist). Untreated cells were used as a control. Twenty-four hours later, the cells were washed with 0.5 mL PBS, and luciferase and β-galactosidase activities were quantified using a luciferase assay kit. Normalized firefly luciferase activity by β-galactosidase activity without treatment was set as 1. WT, wild type; mutated, GATA binding site mutation; underlying uppercase letter, GATA binding sequence; uppercase letter in red, mutated GATA binding sequence.

According to a large epidemiological work, AMPK may positively affect CGD via the transactivation of ABCG5/G8 [22]. Animal studies have shown that ABCG5/G8 play a direct role in the process that leads to bile cholesterol supersaturation, which reduces hepatic cholesterol burden [23]. However, the hepatic cholesterol output and ABCG5/G8 expression remained unchanged between ^AAV2/8^CAV1-injected LD-fed mice, with or without compound c treatment **(**Fig. 2B and D**)**. The ABCG5/G8 genes have long been known as direct targets of the oxysterol receptor LXR [12]. Thus, four ABCG5/G8-LUC chimeric constructs were produced and transiently transfected into HepG2 cells to delineate the cis-acting regions of the ABCG5/G8 gene that are directly responsible for transcriptional regulation by LXR and AMPK. As predicted, the synthetic LXR ligand T0901317 compound (1 µM) induced the expression of luciferase from reporters containing the potential LXR site [12] on ABCG5 gene intron 2 (5’-GGATCACTTGAGGTCA-3’; core similarity = 1.0, matrix similarity = 0.985) or on ABCG8 gene intron 3 (5’-GGATCACCTGAGGTCA-3’; core similarity = 1.0, matrix similarity = 0.935) **(**Fig. 4 A**)**, while these two DNA regions showed a reduced ability to respond to T0901317 in a reporter assay in the presence of an AMPK activator (0.5 mM metformin). Furthermore, in accordance with previous reports[24], AMPK activation impeded LXR-mediated SREBP1c gene transactivation **(**Fig. 4B**)**.

Next, the luciferase activities of reports with the 380 bp ABCG5/ABCG8 intergenic region induced by metformin (0.5 mM) were the same whether T0901317 (1 µM) was present or not **(**Fig. 4 C**)**. Additionally, a report on the analysis of the human ABCG5/ABCG8 intergenic region for potential transcription factor-binding sites revealed that there are only two regulatory elements, transcription enhancer factor 1 (TEF1) and GATA, that are present in mouse and human species [12]. The presence of a GATA site (5’-AGATAA-3’) is particularly interesting because it is known to regulate the expression of ABCG5/G8 [25]. Indeed, AMPK could increase the DNA binding activity of GATA binding protein 4 (GATA4) [26]. We performed binding site mutagenesis combined with luciferase reporter assays to show that GATA site mutation impeded the role of AMPK in ABCG5/G8 gene transcription **(**Fig. 4D**)**. These data implied that LXR and AMPK induced luciferase transactivation by acting on different sites of the ABCG5/G8 gene. AMPK suppressed LXR-dependent ABCG5/G8 gene transcription.

### ***Intraperitoneal***^***AAV2/8***^***CAV1 delivery prevents CGD via the reduction of gallbladder MUC1 expression and the improvement of gallbladder motility.***

**Fig. 5:**
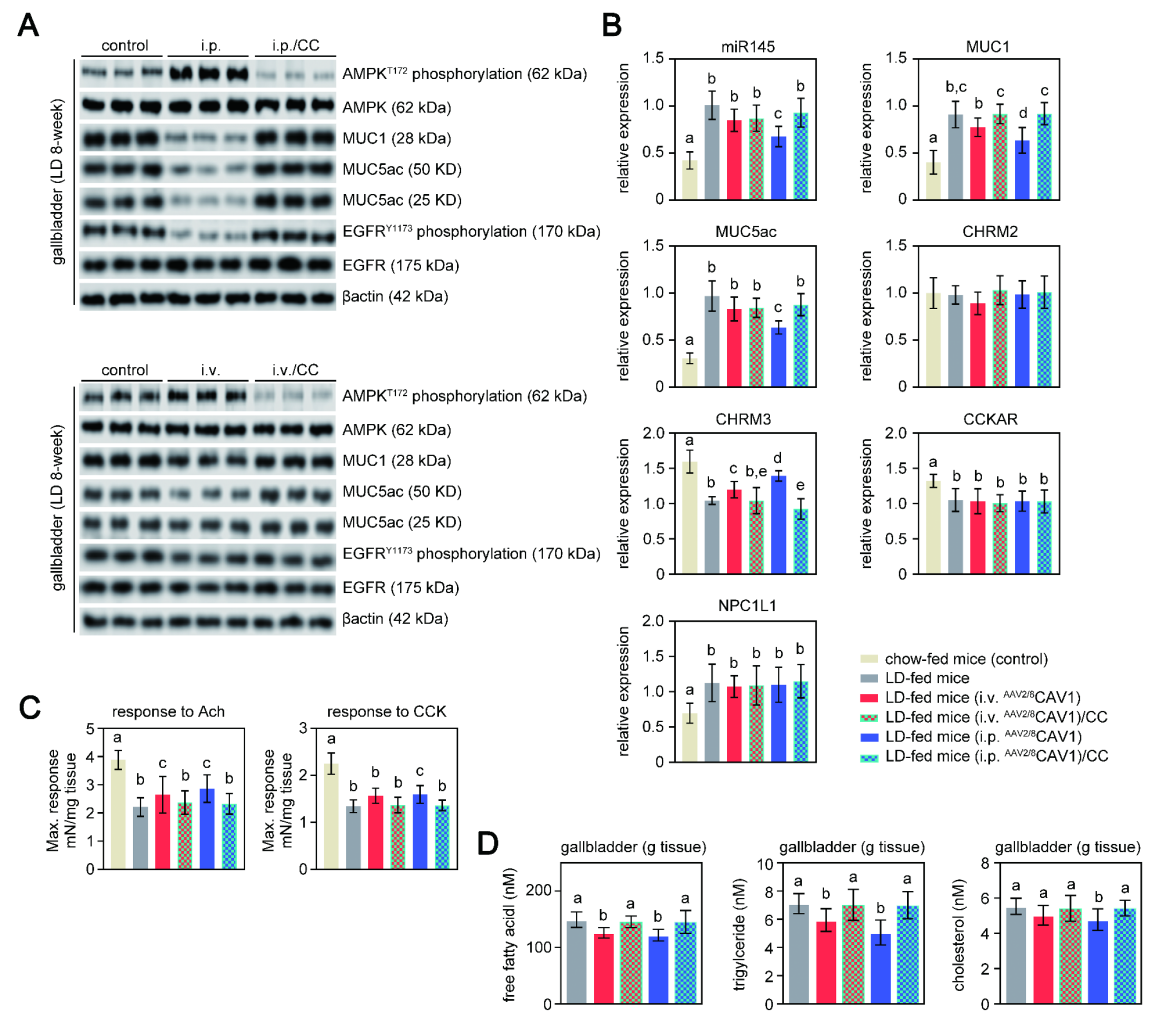
**CAV1-associated gallbladder AMPK activation protects mice from LD-induced CGD via the prevention of gallbladder stasis** Mice were injected (either via the i.v. or i.p. route) with or without ^AAV2/8^CAV1 at 1 × 10^11^ vg/animal and then fed LD following PBS or compound c treatment (8 weeks). n = 13 for each group, LD-fed mice with either i.v. or i.p. administration of ^AAV2/8^CAV1 with or without compound c treatment (10 mg/kg, i.p. injection once a day); n = 9 for LD-fed mice with neither ^AAV2/8^CAV1 administration nor compound c treatment. Chow-fed (8-week) mice (n = 5) were used as a control The letters on each bar are provided for statistical purposes, and different letters indicate significance (*P* < 0.05). If there are no significant differences between two bars, they have the same letter. The individual *P* values are described in Supplementary Table 3. A. Western blotting analysis of AMPK (and its phosphorylation), EGFR (and its phosphorylation), MUC1, and MUC5ac protein expression in the gallbladder of each group of mice. β-actin was used as a loading control. Data are representative of three samples for each protein. T172, the activation marker of AMPK phosphorylation at threonine 172; Y1173, the activation marker of EGFR phosphorylation at tyrosine 1173; i.v., ^AAV2/8^CAV1 injection (1 × 10^11^ vg/mice) via the i.v. route; i.p., ^AAV2/8^CAV1 injection (1 × 10^11^ vg/mice) via the i.p. route; CC, compound c injection (10 mg/kg/day) via the i.p. route. B. qRT‒PCR analysis of CCKAR, CHRM2, CHRM3, miR145-5p, MUC1, MUC5ac, and NPC1L1 expression in the gallbladder of each group of mice. 18 S rRNA was used as an internal control. Data represent the mean ± SD of three independent experiments. CC, compound c injection (10 mg/kg/day) via the i.p. route. C. The contractile force of gallbladder smooth muscle is generated in response to 10 µM acetylcholine or 10 nM cholecystokinin. Data represent the mean ± SD of three independent experiments. Ach, acetylcholine; CCK, cholecystokinin. D. Quantitation of free fatty acids, triacylglycerol, and cholesterol in the gallbladder of each group of mice.

Unlike the i.v. route, i.p. ^AAV2/8^CAV1 administration reduced CGD prevalence in LD-fed mice, although neither of them affected LD feeding-induced bile cholesterol saturation (Fig. 2D **and E**). These data proved that bile cholesterol saturation is required but insufficient for CGD. The formation of cholesterol gallstones is also linked to the accumulation of pronucleating mucins in the gallbladder and its hypomotility, which would enhance the process of bile cholesterol nucleation [7]. It has been reported that epithelial mucin-1 (MUC1) could influence gallbladder motility and the expression of pronucleating MUC5ac [27]. The gel-forming MUC5ac acted as a protective coating to accelerate the appearance of cholesterol monohydrate crystals, whereas mice with epithelial MUC1 deficiency were resistant to CGD due to decreased MUC5ac expression [28]. Only i.p. ^AAV2/8^CAV1 treatment lowered the gallbladder expression of MUC1 and MUC5ac (“MUC1,5ac”) in LD-fed mice, which was attributed to CAV1-associated gallbladder AMPK activation initiating the microRNA-145 (miR145)/MUC1 axis [29, 30] and inhibiting epidermal growth factor receptor (EGFR)/MUC5ac signaling [31] **(**Fig. 5 A **and B)**. As expected, the effect on “MUC1,5ac” expression was greatly reduced after AMPK was turned off **(**Fig. 5 A **and B)**.

Additionally, i.v. or i.p. ^AAV2/8^CAV1 injection rescued the diminished gallbladder smooth muscle contraction force in response to acetylcholine in LD-fed mice, while only i.p. ^AAV2/8^CAV1 administration had a mild effect on damaged gallbladder motility in response to cholecystokinin **(**Fig. 5 C**)**. The difference in gallbladder contraction force between acetylcholine and cholecystokinin stimulation could be a result of restored cholinergic receptor muscarinic 3 (CHRM3) expression in the gallbladder of LD-fed mice treated with ^AAV2/8^CAV1 but who retained downregulated gallbladder cholecystokinin receptor (CCKAR) expression **(**Fig. 5B**)**. LD feeding and/or ^AAV2/8^CAV1 treatment did not affect gallbladder cholinergic receptor muscarinic 2 (CHRM2) expression **(**Fig. 5B**)**. Triglyceride-associated lipid stress contributes to muscarinic receptor damage [32], whereas excessive intracellular cholesterol accumulation impairs CCK signaling [7]. ^AAV2/8^CAV1 treatment reduced gallbladder free fatty acid and triglyceride contents in 8-week LD-fed mice **(**Fig. 5D**)**, but only i.p. injection of ^AAV2/8^CAV1 obviously diminished the cholesterol levels in gallbladder cells **(**Fig. 5D**)**. However, the cholesterol content of the gallbladder was comparable between the control and i.v. ^AAV2/8^CAV1-treated mice, possibly due to the increased gallbladder MUC1 expression of mice in response to LD feeding, which had a role in the gallbladder absorption of cholesterol from bile **(**Fig. 5 A and 5B**)**. Notably, the expression of gallbladder NPC1L1 was similar between LD-fed mice with or without ^AAV2/8^CAV1 treatment **(**Fig. 5B**)**. These findings suggest that the primary mechanism by which i.p. ^AAV2/8^CAV1 administration protects against CGD by preventing hyperproduction of gallbladder MUC1.

## Discussion

CGD occurs or recurs frequently, making it one of the most expensive gastrointestinal problems to treat [7]. While laparoscopic cholecystectomy is considered the gold standard for gallstone removal, bile duct injuries or long-term gastrointestinal abnormalities have been reported following surgery. Novel therapeutics are vigorously being sought to treat CGD. Laparoscopy combined with choledochoscopic lithotomy is a viable surgical option for CGD patients who still have normal gallbladder function [33]. However, CGD patients with gallbladder hypomotility might not be good candidates for gallbladder preservation operations due to a high recurrence rate of gallstones. These data suggest that safeguarding gallbladder motility will aid in the development of novel CGD treatment strategies.

Previous work by Tharp et al. [34] using CGD mice found that maintaining gallbladder physiological function via gallbladder long-chain fatty acid transporter 2 (FATP2) deletion by systemic i.v. injection of AAV-delivered short hairpin RNA (shRNA) is sufficient to prevent gallstone formation, even in the presence of supersaturated bile cholesterol. Here, we found that the i.v. ^AAV2/8^CAV1 injection elevated hepatic CAV1 expression in mice before or after LD feeding. However, gallbladder CAV1 expression was marginally affected by ^AAV2/8^CAV1 treatment via the i.v. route. The transgene sequence size may influence AAV transfer efficiency [35, 36]; therefore, the lower efficiency of AAV-assisted CAV1 gene delivery into gallbladder tissues in our work compared with that of Tharp et al. [34] might be attributed to the size of the CAV1 gene coding sequence (CDS) being significantly higher than that of shRNA targeting FATP2. It has been described that local AAV delivery methods such as i.p. injection is more efficient than systemic i.v. administration [11]. We observed that ^AAV2/8^CAV1 injection via the i.p. route led to an enhanced transduction efficiency in the gallbladder collected from chow-fed or 8-week LD-fed CGD mice compared to the i.v. route, while hepatic CAV1 protein expression levels were similar between mice injected i.p. or i.v. ^AAV2/8^CAV1 delivery. In addition, a minor change in CAV1 expression in the intestine of mice with either i.p. or i.v. ^AAV2/8^CAV1 treatment was also similar to a previous report [14].

We previously demonstrated that mice with global CAV1 knockout were prone to developing CGD [34]. In this work, obvious CAV1 overexpression was observed in the liver after i.v. ^AAV2/8^CAV1 treatment or in both the liver and the gallbladder through i.p. ^AAV2/8^CAV1 injection allowed us to distinguish the role of CAV1 in gallbladder motility and its contribution to the prevention of CGD, independent of changes in bile cholesterol saturation. Either i.v. or i.p. ^AAV2/8^CAV1 injection reduced LD-fed-induced hepatic triglyceride and cholesterol accumulation via the increase in CAV1 protein levels. A potential mechanism for the alleviation of hepatic lipid abnormalities in LD-fed mice following ^AAV2/8^CAV1 treatment is associated with CAV1-mediated AMPK activation to downregulate SREBP1c and SREBP2 [20, 24]. We also found that AMPK activation impeded LXR-ABCG5/G8, but it itself increased ABCG5/G8 expression in the liver to promote cholesterol entering the bile, which prevented liver cholesterol accumulation to alleviate LD-induced liver lipid toxicity. We noticed that, unlike stimulation by LXR via intron binding [12], AMPK-mediated ABCG5/G8 gene transactivity may be dependent on a conservative GATA site in the intergenic region between the two genes [12, 26]. However, hepatic AMPK-ABCG5/G8 signaling activation also elevated the biliary CSI to increase CGD risk, which might be why LD-fed mice with or without ^AAV2/8^CAV1 injection exhibited a similar elevated biliary CSI.

The major difference between LD-fed mice with i.v. and i.p. ^AAV2/8^CAV1 delivery resulted in obvious CAV1-associated gallbladder AMPK activation and LD-induced gallbladder dysmotility. The expression of epithelial MUC1 and the gel-forming MUC5ac, which have been identified as the predominant pro-nucleating factors for CGD [27, 28, 31], was also downregulated in LD-fed mice following i.p. injection of ^AAV2/8^CAV1. Gallbladder hypersecretion of “MUC1,5ac” and its abnormal emptying are two independent risk factors for gallbladder stasis and CGD [7]. However, mice transgenic for the human MUC1 gene displayed increased MUC5ac production and impaired gallbladder emptying function via increased gallbladder cholesterol absorption in response to LDs, which implied that MUC1 provides a bridge between gallbladder hypomotility and pronucleating MUC5ac hyperproduction [27]. Gallbladder AMPK activation induced by i.p. ^AAV2/8^CAV1 injection was responsible for the reduction in “MUC1,5ac” expression. miR145, a downstream effector of AMPK signaling [37], could bind to the 3’ untranslated region of the MUC1 gene [29, 30], thereby linking the reduction of MUC1 with AMPK signaling. Furthermore, it is well known that epithelial MUC1 on the cell surface promotes EGFR activation [38] and that the epidermal growth factor (EGF)/EGFR axis induces MUC5AC expression [31], whereas AMPK inhibits EGFR activity [39]. Our experimental data proved that i.p. ^AAV2/8^CAV1 injection induced gallbladder AMPK activation and MUC5ac expression downregulation, which might occur by modulating EGFR phosphorylation. However, whether i.p. ^AAV2/8^CAV1 injection-induced gallbladder MUC1 reduction contributes to AMPK-mediated downregulation of EGFR/MUC5ac signaling remains unknown.

Furthermore, meal-stimulated gallbladder emptying occurs as a result of gallbladder contraction mediated by CCK and/or acetylcholine signaling [40], which, when weakened by gallbladder lipid accumulation due to LDs, may provide the retention time for bile cholesterol nucleation [7]. Acetylcholine contracts the gallbladder through CHRM2 and CHRM3 [41], while CCK regulates gallbladder motility via CCKAR [7]. CHRM3 and CCKAR expression in the gallbladder of LD-fed mice was lowered, while CHRM2 expression was unaffected. Here, by using LD-fed mice, we found that either i.v. or i.p. ^AAV2/8^CAV1 delivery could improve gallbladder cholinergic responsiveness, which might be attributed to restored gallbladder CHRM3 expression by the hepatic CAV1/AMPK axis contributing to whole-body lipid homeostasis. However, only i.p. ^AAV2/8^CAV1 administration partially repaired the responsiveness of the gallbladder to CCK, but neither it nor i.v. ^AAV2/8^CAV1 administration had any influence on the reduced CCKAR or on the increased NPC1L1 in the gallbladder of LD-fed mice. The work of Wang et al. [27] provides evidence that MUC1 increases gallbladder cholesterol uptake from supersaturated bile and impairs its motility. Cholesterol accumulation decouples phospholipase C signaling from CCKAR, thereby impairing physiological gallbladder motility [7]. In addition, gallbladder cells do not have the ability to assemble lipoproteins and transport them into plasma [27]. Therefore, the discrepancy in gallbladder CCK responsiveness between i.v. and i.p. ^AAV2/8^CAV1 treatment was attributable to the i.p. route of ^AAV2/8^CAV1 injection inhibits MUC1-related gallbladder cholesterol absorption from the bile via the gallbladder CAV1/AMPK axis. Further work elucidating the underlying mechanisms of LD feeding on the reduced expression of CHRM3 and CCKAR in the gallbladder may support advancements in the prevention and treatment of CGD.

## Study strengths and limitations

In this study, the i.v. and i.p. injection routes of ^AAV2/8^CAV1 have been compared in mice for their ability to prevent CGD from the LD. And the data presented in this work suggested that i.p. ^AAV2/8^CAV1 injection is superior for the transduction of gallbladder cells versus i.v. injection. And i.p. injection of ^AAV2/8^CAV1 partially protected LD-fed mice from developing CGD via improved gallbladder stasis as well as AMPK signaling and reduced MUC1 expression. However, the limitations of this study should be mentioned. Since these experiments were performed on a wild-type background, we cannot conclusively rule out secondary effects on the possible upregulation of endogenous CAV1 following AAV delivery. Future studies to deliver Cre recombinase or fluorescent reporter genes are needed to determine the efficiency of gallbladder transduction and more rigorously compare the i.p. to i.v. route of AAV administration.

## Conclusion

In summary, these data might lead to the conclusion that CGD prevention by i.p. ^AAV2/8^CAV1 injection improves gallbladder stasis by activating gallbladder AMPK signaling. When compared to the same vector administered via i.v. infusion, ^AAV2/8^CAV1 i.p. injection leads to greater CAV1 expression levels in the gallbladder and lower CGD incidence in LD-fed mice via reduction of gallbladder MUC1 expression and enhancement of gallbladder motility. This will be a great tool for performing preclinical functional studies on keeping the gallbladder working normally to prevent CGD.

## Electronic supplementary material

Below is the link to the electronic supplementary material.

Supplementary Material 1

Supplementary Material 2

Supplementary Material 3

Supplementary Material 4

Supplementary Material 5

Supplementary Material 6

## Data Availability

The data that support the findings of this study are available in the supplementary data in this article.

## Declarations

### Authors’ information (optional)

Sha Li, Hongtan Chen, Xin Jiang, Fengling Hu and Guoqiang Xu.

Department of Gastroenterology.

The First Affiliated Hospital, Zhejiang University School of Medicine.

79 Qingchun Road.

Hangzhou, Zhejiang, 310,006, China.

Yiqiao Li.

Urology & Nephrology Center, Department of Nephrology.

Zhejiang Provincial People’s Hospital and Hangzhou Medical College Affiliated People’s Hospital.

158 Shangtang Road.

Hangzhou 310,014, Zhejiang, China.

## Abbreviations

DAPI: 2-(4-Amidinophenyl)-6-indolecarbamidine
AAV2/8: AAV2 inverted terminal repeat DNA combined with the AAV8 capsid
AAV: adeno-associated virus
AMPK: adenosine monophosphate-activated protein kinase
ABCG5: ATP binding cassette subfamily G member 5
BSEP: bile salt export pump
CAV1: caveolin-1
CGD: cholesterol gallstone disease
CSI: cholesterol saturation index
CHRM2: cholinergic receptor muscarinic 3
CHRM3: cholinergic receptor muscarinic 3
CDS: coding sequence
CYP7a1: cytochrome P450 7A1
EGFR: epidermal growth factor receptor
EGF: epidermal growth factor
GATA4: GATA binding protein 4
i.v.: intravenous
LD: lithogenic diet
LXR: liver X receptor
i.p.: local intraperitoneal
miR145: microRNA-145
MUC1: mucin-1
MUC5ac: mucin-5ac
MDR2: multidrug resistance protein 2
MDR2: multidrug resistance protein 2
MRP2: multidrug resistance protein 2
NAFLD: nonalcoholic fatty liver disease
NT: nucleation time
SREBP1c: sterol-regulatory-element-binding protein-1c
SREBP2: sterol-regulatory-element-binding protein-2
TEF1: transcription enhancer factor 1
vg: viral genomes
type C1-like intracellular cholesterol transporter 1: niemann-pick disease
