# Supplementary figures and images for: Adeno-associated virus-based caveolin-1 delivery via different routes for the prevention of cholesterol gallstone formation
Source: Lipids Health Dis. 2022 Oct 27;21:109. doi: 10.1186/s12944-022-01718-7 (PMC9609467; doi:10.1186/s12944-022-01718-7)

# Supplementary Figure 1

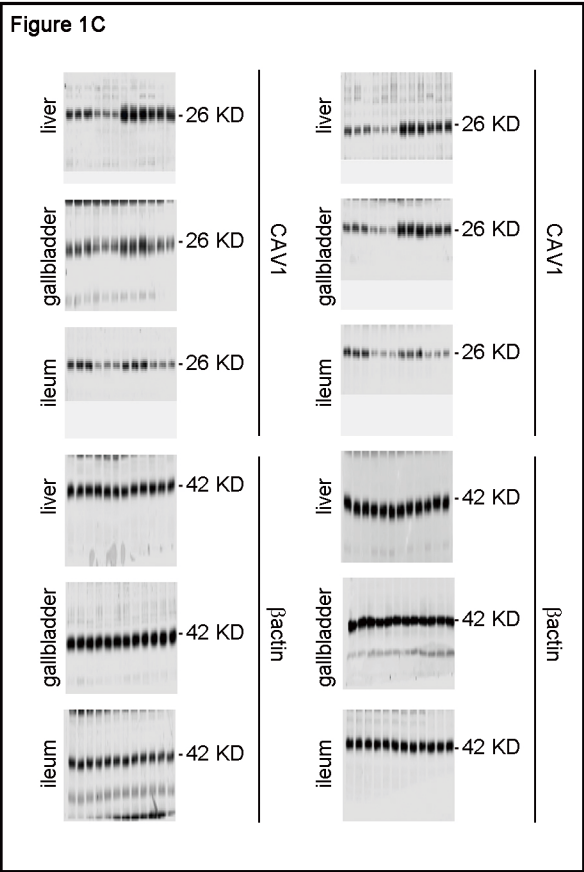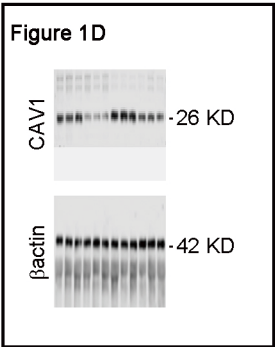

Supplement: Supplementary file 1 — Supplementary Material 1 [file 12944_2022_1718_MOESM1_ESM.pdf]

# Supplementary Figure 2

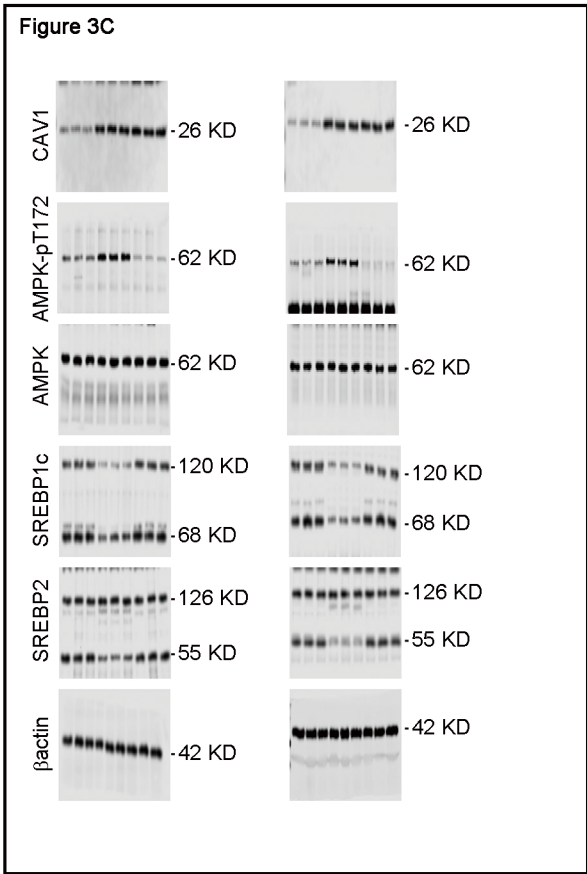

Supplement: Supplementary file 2 — Supplementary Material 2 [file 12944_2022_1718_MOESM2_ESM.pdf]

Supplementary Figure 3

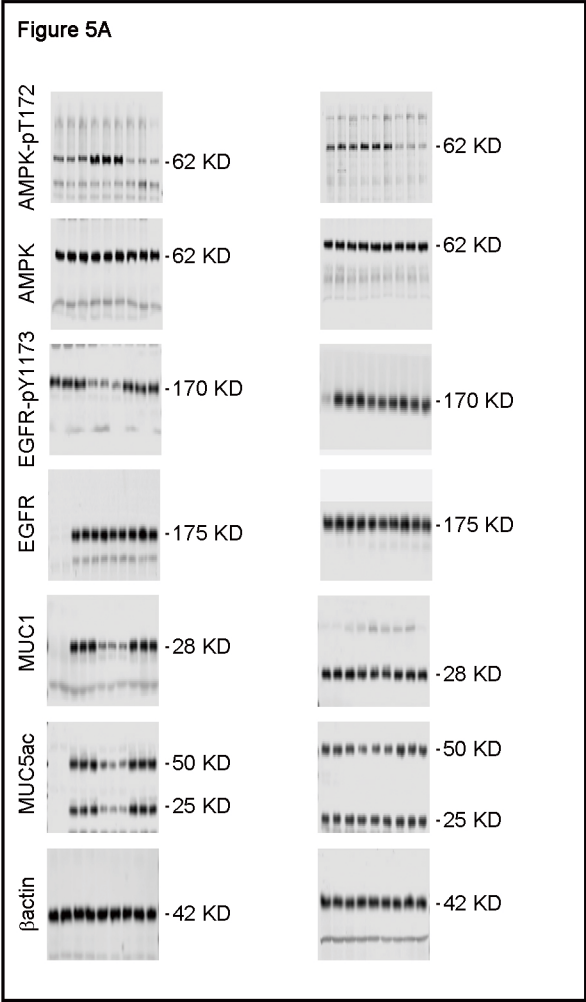

Supplement: Supplementary file 3 — Supplementary Material 3 [file 12944_2022_1718_MOESM3_ESM.pdf]
